# High prevalence of fluconazole resistant *Candida tropicalis* among candiduria samples in China: An ignored matter of concern

**DOI:** 10.3389/fmicb.2023.1125241

**Published:** 2023-03-02

**Authors:** Xin Fan, Clement K. M. Tsui, Xi Chen, Peng Wang, Zhen-jia Liu, Chun-xia Yang

**Affiliations:** ^1^Department of Infectious Diseases and Clinical Microbiology, Beijing Institute of Respiratory Medicine and Beijing Chao-Yang Hospital, Capital Medical University, Beijing, China; ^2^National Centre for Infectious Diseases, Tan Tock Seng Hospital, Singapore, Singapore; ^3^Lee Kong Chian School of Medicine, Nanyang Technological University, Singapore, Singapore; ^4^Division of Infectious Diseases, Faculty of Medicine, University of British Columbia, Vancouver, BC, Canada

**Keywords:** *Candida tropicalis*, azole resistance, *ERG11*, candiduria, invasive infections

## Abstract

**Introduction:**

The rapid rise of azole resistance in *Candida tropicalis* causing invasive infections has become a public health concern; however, the prevalence of resistant isolates in urine samples was not well studied, because the clinical significance of candiduria was not unambiguous due to possible host colonization.

**Methods:**

We performed a 12-year laboratory-based surveillance study of *C. tropicalis* causing either invasive infection or candiduria and studied their susceptibility profiles to common antifungal drugs. The complete coding domain sequence of the *ERG11* gene was amplified in all fluconazole resistant isolates, and aligned with the wild-type sequence to detect nucleotide mutations.

**Results:**

A total of 519 unique *C. tropicalis* strains isolates, 69.9% of which were isolated from urine samples and remaining 30.1% were invasive strains. Overall, 16.5% isolates were confirmed to be resistant to fluconazole, of which 91.9% were cross-resistant voriconazole. Of note, at the beginning of surveillance (2010–2011), the fluconazole resistance rates were low in both candiduria and invasive groups (6.8% and 5.9%, respectively). However, the resistant rate in the candiduria group significantly increased to 29.5% since 2012–2013 (*p* = 0.001) and stayed high since then, whilst the resistance rate in the invasive group only showed a gradually increasing trends till 2021 (*p* > 0.05). Sequence analysis of *ERG11* from fluconazole-resistant strains revealed the prevalence of A395T/W mutations were relatively low (16.7%) in the beginning but reached 87.5–100% after 2014. Moreover, the A395W heterozygous mutation isolates became predominant (>60% of resistant strains) after 2016, and indeed isolates carrying corresponding amino acid substitution (Y132F) was highly resistant to fluconazole with MIC_50_ exceeded 256 μg/ml.

**Conclusion:**

Our study revealed high azole resistant rate in candiduria with its increasing trends observed much earlier than stains causing invasive infections. Given antimicrobial resistance as a critical “One Health” issue, the emergence of antifungal resistance in *Candida* species that are common commensal colonizers in the human body should be concerned.

## Introduction

*Candida tropicalis* is a globally distributed commensal yeast and is one of the most common pathogenic non-*Candida albicans* species causing human infections (Kullberg and Arendrup, 2016; Pappas et al., 2018). Owning to the new technological advances in modern medicine and accessibility to therapies and interventions that impair the immune system, such as chemotherapy and immunotherapy for cancer, and solid organ transplantation, the incidence of invasive fungal infections has increased (Kullberg and Arendrup, 2016; Bongomin et al., 2017; McCarty et al., 2021). The life-threatening invasive infections, such as candidemia, fungal meningitis and other internal organs infections, are of most clinical concern (Kullberg and Arendrup, 2016; Pappas et al., 2018; McCarty et al., 2021).

Of note, the public health threat and challenge of invasive fungal diseases are compounded by the rapid emergence of antifungal resistance. In China, there are increasing concerns raised by high azole resistant rates in *C. tropicalis* isolates causing invasive infections (Fan et al., 2017; Guo et al., 2017; Wang et al., 2021). Various levels of a surveillance program in identifying the resistance rate have been performed to guiding clinical antifungal treatment for invasive infections. However, according to a previous study conducted during 2012–2013 and 2016–2017 in our hospital (a tertiary teaching hospital in Beijing), the rate of azole resistance among invasive *C. tropicalis*, was only 5.1% (Fan et al., 2020), which was much lower than nationwide data (12.8 to 22.2%) in China (Fan et al., 2017; Wang et al., 2021).

Candiduria is commonly observed in hospitalized patients. Major risk factors for candiduria are diabetes mellitus, indwelling urinary catheters, use of broad-spectrum antibiotics, urinary obstruction, and admission to intensive care units (Kauffman et al., 2000; Sobel et al., 2011; Odabasi and Mert, 2020; Chotiprasitsakul et al., 2021; Shirvani and Fattahi, 2021). While determining the clinical significance of candiduria is always challenging, because it may reflect either *Candida* colonization or infection (Sobel et al., 2011; Alfouzan and Dhar, 2017). As the at-risk population continues to expand, *Candida* species have been increasingly reported causing nosocomial urinary tract infections (UTIs) (Kauffman et al., 2000; Alfouzan and Dhar, 2017; Trzesniewska-Ofiara et al., 2022). More importantly, in our hospital’s routine clinical practice and report, we observed that the azole resistant *C. tropicalis* isolates were more frequently detected in urine specimens. While antifungal resistance in candiduria was not well-documented in China, as most attention was driven towards invasive fungal diseases, and urine samples have been excluded from the nationwide surveillances (Wang et al., 2012; Fan et al., 2017; Wang et al., 2021).

In this study, we investigate the antifungal resistance of *C. tropicalis* causing invasive infections and in candiduria over an 12-years period, and we characterize the *ERG11* mutations in all azole resistant strains. The study provides important information on the prevalence of fluconazole resistant *Candida tropicalis* among candiduria and invasive samples, which are useful for patient care and antifungal stewardship program.

## Materials and methods

### Study design

This was a 12-year (1 January 2010 to 31 December 2021) laboratory-based surveillance study of *C. tropicalis* in Beijing Chaoyang Hospital, a tertiary teaching hospital with 1,400 inpatient beds. The strains causing invasive infections per inclusion criteria as described before (Wang et al., 2012), and recovered from urine samples with clone count >10^3^ cfu/ml (Kauffman, 2014; Lima et al., 2017) were included in this study. Isolates from blood, normally sterile body fluids, including ascitic fluid, bile and pleural fluid, abscesses, tissue samples, central venous catheter (CVC) tips and bronchoalveolar lavage fluid (BALF) needed to be the agent of invasive infection. Duplicated isolates from the same body site of a patient at different times were excluded. For each yeast isolate, clinical information was collected into a standard case report form, including the patient’s age and gender, inpatient or outpatient status, and the ward location, date of sample collection, specimen type, body site of isolation and initial azole susceptibility. All the isolates were identified by matrix-assisted laser desorption/ionization-time of flight mass spectrometry (Vitek MS; bioMérieux, Marcy l’Etoile, France).

### Antifungal susceptibility testing

All strains were tested for fluconazole and voriconazole susceptibility using disk diffusion method following Clinical and Laboratory Standards Institute (CLSI) document (Clinical and Laboratory Standards Institute, 2022). Minimum inhibitory concentrations (MICs) of nine antifungal agents were re-measured using Sensititre YeastOne™ YO10 methodology (Thermo Scientific, Cleveland, OH, United States) for strains resistant or susceptible-dose dependent (SDD) for either fluconazole or voriconazole by initial disk diffusion method (Clinical and Laboratory Standards Institute, 2022). The nine antifungal agents tested included four azoles (fluconazole, voriconazole, itraconazole and posaconazole), three echinocandins (caspofungin, micafungin and anidulafungin), 5-flucytosine and amphotericin B. Current available clinical breakpoints (CBPs) or epidemiological cut-off values (ECVs) were used for interpretation of susceptibility results (Clinical and Laboratory Standards Institute, 2022). The quality-control strains were *Candida parapsilosis* ATCC 22019 and *Candida krusei* ATCC 6258 as recommended by CLSI document (Clinical and Laboratory Standards Institute, 2022).

### Sequencing of *ERG11* gene

Prior to DNA extraction, isolates were sub-cultured onto Sabourauds dextrose agar for 24 h at 35°C. DNA extraction was performed using a glass bead and heating-assisted QIAamp DNA mini kit (Qiagen, Hilden, Germany) method. The complete coding domain sequence of the *ERG11* gene was amplified in all fluconazole resistant isolates, and PCR products were sequenced using primer pairs as described previously (Vandeputte et al., 2005). The chromatograms were visually checked and examined for heterozygosity. Then nucleotide sequences were aligned to the wild-type *ERG11* gene of *C. tropicalis* MYA-3404 (GenBank accession no. XM_002550939.1) to detect nucleotide mutations using CLC SEQUENCE VIEWER software (version 8.0, Qiagen Bioinformatics, Aarhus, Denmark).

### Statistical analysis

All statistical analyses were performed using SPSS software version 24.0 (SPSS, Inc., IL, United States). Comparisons of continuous variables were performed using the *χ*^2^ test or Fisher’s exact test, as appropriate. *p*-value less than 0.05 was considered statistically significant.

## Results

### Distribution of specimen type and patient characteristics

A total of 519 *C. tropicalis* strains were isolated from 485 patients during the 12-year surveillance period, of which 363 (69.9%) strains were isolated from urine samples and the remaining 156 (30.1%) were from invasive infection (IVF) samples. Amongst 485 patients enrolled, 25 (5.2%) had *C. tropicalis* detected simultaneously in urine and IVF samples. Most of these patients (72.0%, 18/25) were from ICU. The most common specimen of invasive infection was ascitic fluid and BALF, both accounting for 8.3%, respectively (Supplementary Table 1).

**Figure 1:**
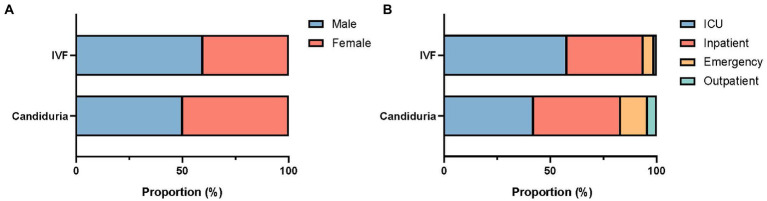
Gender **(A)** and patient location **(B)** composition of patients with candiduria and invasive infections (IVF).

There was a difference in patients’ gender composition between urine and IVF groups, with proportion of female patients higher in urine group (49.9%) than in IVF group (40.3%) (*p* = 0.049, Figure 1A). Of candiduria patients, 42.3 and 40.4% were from ICUs and general inpatients, respectively, 12.8% were from the emergency department and 4.5% from outpatients. In comparison, more patients (85/149, 57.0%) with invasive infections were from ICUs (*p* = 0.002), with less proportion (36.2%) from general inpatients, and 6.7% were from the outpatient/emergency department setting (Figure 1B). Besides, 22.4% (85/363) of *C. tropicalis* candiduria patients also had bacterial pathogens detected in their urine samples.

### Antifungal susceptibilities

Of all the 519 isolates in this study, only 121 isolates with the zone diameters less than 17 mm and the MICs of nine antifungals were measured. Based on CLSI M27M44S breakpoints and epidemiological cutoff value (ECV) (Clinical and Laboratory Standards Institute, 2022), no echinocandins-resistant isolates were detected, and all isolates were susceptible (wild type) to amphotericin B (MIC≤2 μg/ml). Only one 5-flucytosine resistant strain was detected (MIC = 64 μg/ml). For azoles, 16.5% (86/519) isolates were resistant to fluconazole, of which 91.9% (79/86) were cross-resistant voriconazole and all remaining 8.1% (7/86) isolates were intermediate to voriconazole. No strain resistant to voriconazole but susceptible to fluconazole was detected.

**Figure 2:**
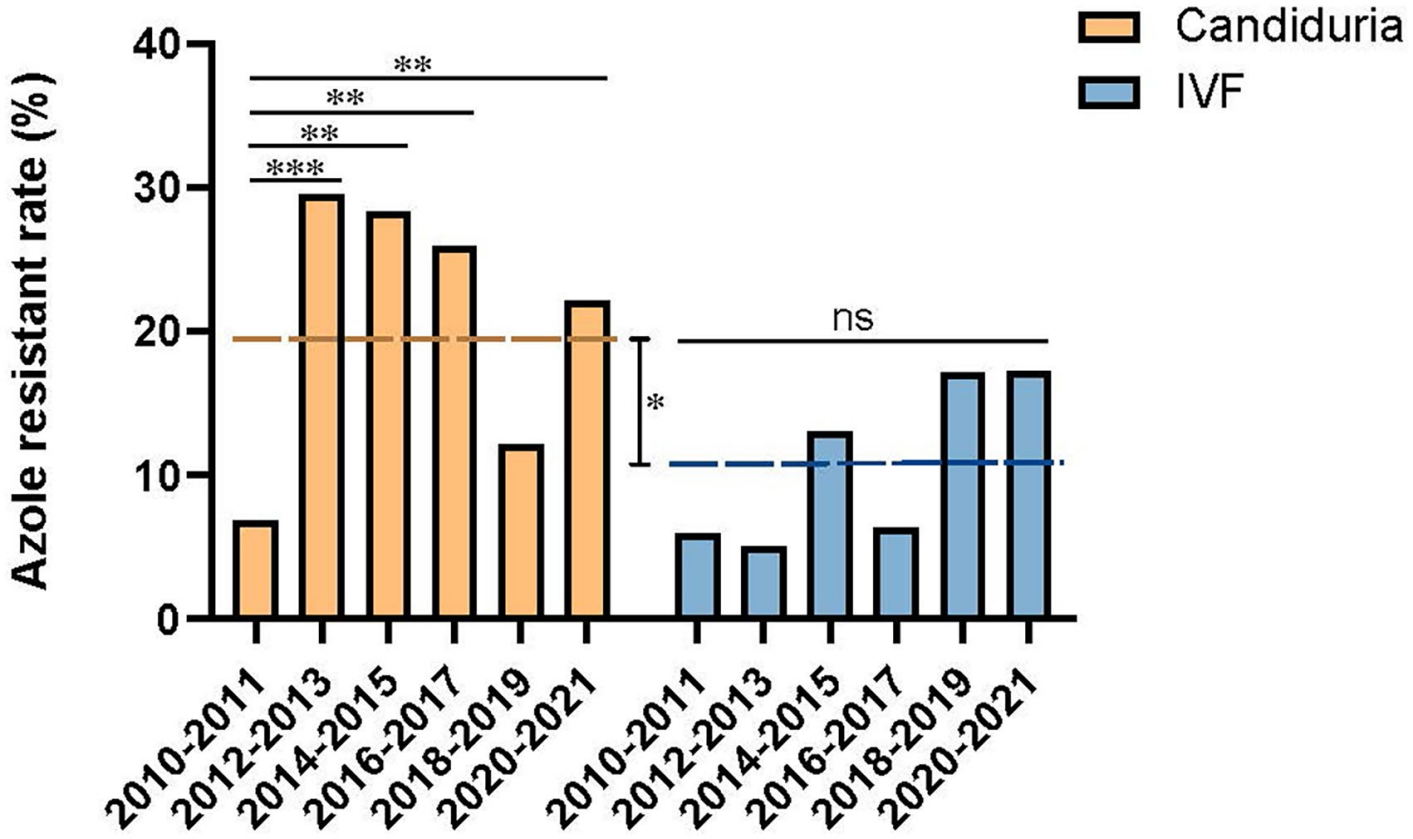
Temporal changes of azole resistance of *C. tropicalis* isolated from candiduria and invasive infection (IVF) patients. The dashed line indicates the overall fluconazole resistance rates of latter nine surveillance years’ period (2012–2021). *p < 0.05, **p < 0.01, ***p < 0.001, and ns, not significance.

The overall fluconazole resistant rate was 19.0% (69/363) in candiduria isolates, significantly higher than that of isolates causing invasive infections (10.9%, 17/156) (*p* = 0.023). In addition, at the beginning of our surveillance (2010–2011), the fluconazole resistance rates were similarly low in isolates from candiduria and invasive infection strains (6.8%, 5/74 in urine vs. 5.9%, 1/17 in IVF group). However, the rising of fluconazole resistance rate in candiduria group was already notable in the second surveillance period, elevated from 6.8 to 29.5% in 2012–2013 (*p* = 0.001) (Figure 2). In contrast, the resistance rate in invasive group only showed a gradually increasing trends till 2018–2019 (14.3%, *p* > 0.05), but then reached 17.2% (5/24) in 2020–2021 (Figure 2).

### Missense mutations in the *ERG11* gene

**Figure 3:**
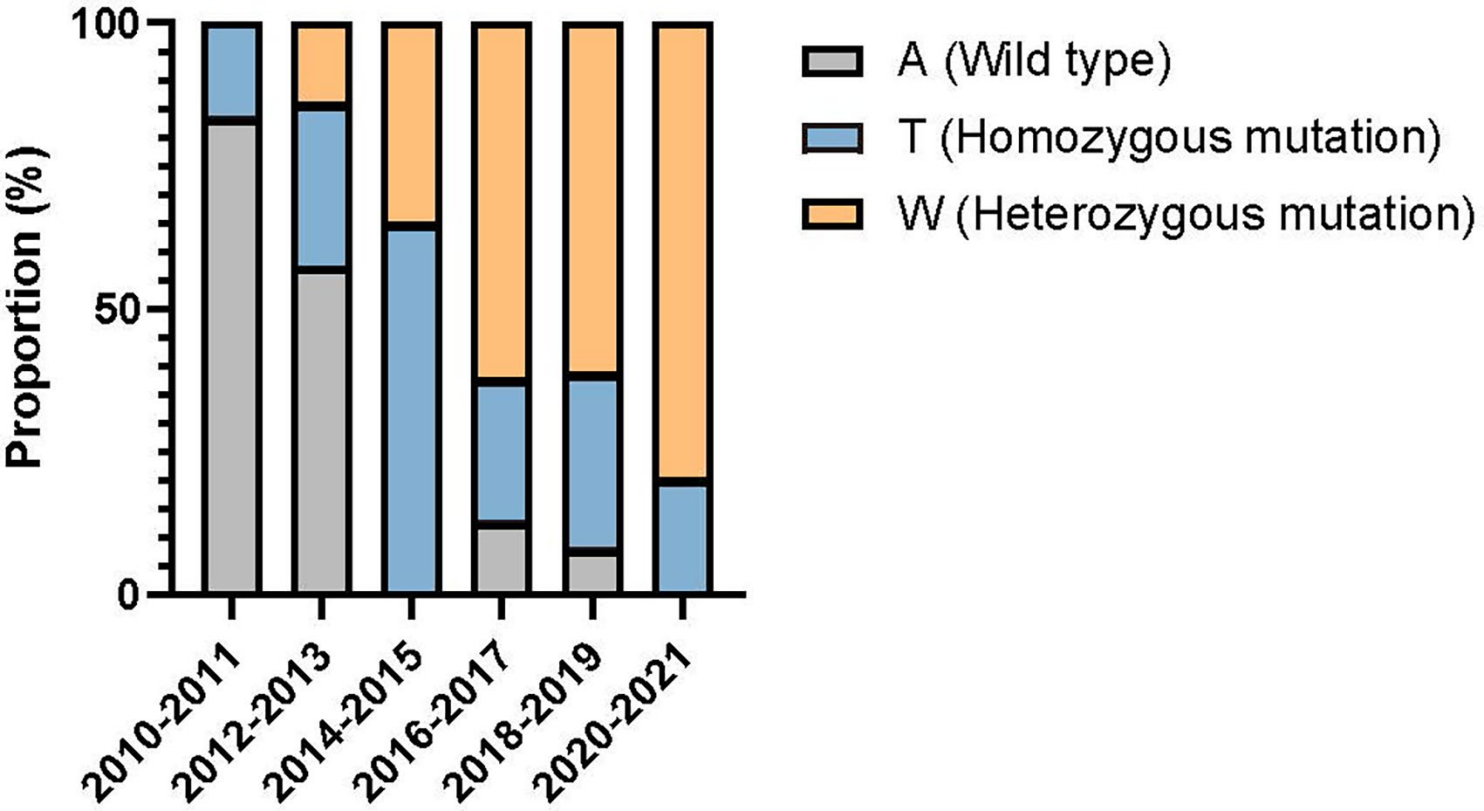
Temporal shift in the composition of single nucleotide polymorphisms at position 395 of *ERG11* gene in 86 fluconazole-resistant strains.

Compared with the wild-type *ERG11* sequence of *C. tropicalis* MYA-3404, four missense mutations, T374C (V125A), A395T/W (Y132F), C461T/Y (S154F), and T769C/Y (Y257H) that have been well-characterized in previous studies (Fan et al., 2020; Keighley et al., 2022; Leepattarakit et al., 2022), were detected in 86 fluconazole-resistant strains. No novel missense mutation was identified. Strains bearing A395T/W and C461T/Y double mutations were the most common and were detected in 67 of 86 (77.9%) isolates. Two other A395T/W single mutation strains were detected. As previous studies have demonstrated that C461T/Y mutation in *ERG11* does not influence fluconazole susceptibility and A395T/W is the predominant mechanism responsible for azole resistance in *C. tropicalis* (Fan et al., 2019), we further analyzed the temporal distribution of mutations for A395T/W in *ERG11* gene. We found that most of the fluconazole resistance phenotype (5/6, 83.3%) at the beginning of surveillance (2010–2011) were not attributed to A395T/W mutations; however, isolates with these mutations increased rapidly, and reached 87.5–100% amongst all resistant strains after 2014 (*p* < 0.01) (Figure 3). Moreover, the proportion of isolates with A395W heterozygous mutations rapidly became predominant since 2016 (>61.5%) (Figure 3). Of note, both A395T/W heterozygous and homozygous mutation strains were highly resistant to fluconazole, with their MIC_50_ exceeded 256 μg/ml. Strains carrying mutation T374C and/or T769C/Y were rare; only one isolate bearing T374C mutation while three isolates having the T769C/Y mutations (Table 1). These two mutations have been linked to azole resistance previously (Fan et al., 2019). In addition, 12 fluconazole resistance isolates carrying *ERG11* wild type sequence were reported in this study, ten of which were detected in the first 4 years of the surveillance period.

**Table 1:** Azole target gene *ERG11* genotypes among 86 fluconazole resistant *Candida tropicalis* isolates.

Nucleotide mutation position in *ERG11* gene				Number of isolates	Proportion
374	395	461	769		
Reference wild-type sequence (GenBank accession no.: XM_002550939.1)					
T	A	C	T	–	–
Clinical strains sequences					
T	**W**	**T**	T	34	39.5%
T	**T**	**T**	T	27	31.4%
T	**W**	**Y**	T	6	7.0%
T	A	C	**C**	2	2.3%
**C**	A	C	T	1	1.2%
T	A	**Y**	T	1	1.2%
T	T	C	T	1	1.2%
T	**W**	C	T	1	1.2%
T	**W**	**Y**	**Y**	1	1.2%
T	A	C	T	12	14.0%

Bold and underlined labelled nucleotides were mutation positions identified.

## Discussion

The overall isolation rate of *C. tropicalis* amongst candidiasis was approximately 9% based on a global multicenter surveillance study, but it was more prevalent in the Asia-Pacific region, accounting for 14% of cases (Pfaller et al., 2019). Moreover, the mortality rate of invasive *C. tropicalis* infections can reach 61–77.8%, which is significantly higher than that of other *Candida* species (Munoz et al., 2011; Montagna et al., 2013). According to the latest fungal priority pathogens list developed by WHO, *Candida tropicalis* is classified as a “high priority” fungal pathogen, ranked next to the highest “critical priority” (World Health Organization, 2022). The main reason may be due to the relatively lower resistance rate of *C. tropicalis* to azoles, including fluconazole, itraconazole, voriconazole and posaconazole, ranging from 0 to 20% in many countries and regions (Pfaller et al., 2019; Denning, 2022). However, azole resistance in *C. tropicalis* has raised greater public concerns in China. According to a nationwide surveillance study including over 80 participating hospitals, the rate of azole resistance in *C. tropicalis* causing invasive infections has reached 31.8% in 2018 in China (Wang et al., 2021). By contrast, the azole resistance rate of invasive *C. tropicalis* strains in our hospital remained lower than the national surveillance data, both noted in our previous report (Fan et al., 2020) and in this study. A multicenter surveillance of invasive fungal diseases in Beijing (where our hospital was located) also reported low azole resistant rates in *C. tropicalis* (9.1%) (Guo et al., 2017), which was in consistent to our findings. These geographic variations indicated the importance of carrying out regional and institutional surveillance for guiding patient management as well as antifungal stewardship program.

From a clinical perspective, the presence of candiduria is not considered as strong evidence for urinary infection due to the possibilities of colonization or contamination, and antifungal therapy is not recommended for asymptomatic patients with candiduria (Alfouzan and Dhar, 2017; Odabasi and Mert, 2020). As a result, many previous nationwide surveillance studies excluded isolates from the urine samples to ensure proper clinical interpretation of results (Wang et al., 2012; Fan et al., 2017; Guo et al., 2017; Wang et al., 2021), thus antifungal resistance amongst candiduria samples remained unclear or even underestimated. However, according to the “One Health” perspective, combating antimicrobial resistance do not only rely on rational use of antimicrobials in clinical treatment, but also require boarder awareness and actions in curbing emerging of resistance in natural environment and human commensal reservoirs (Brinkac et al., 2017; Hernando-Amado et al., 2019). For instance, a previous study found that the wide application and persistence of azoles in the environment contributed to the development of azole resistance in non-target organisms such as, *Aspergillus fumigatus* (Burks et al., 2021) and the resistant isolates were likely to spread from the environment to the human hosts (Snelders et al., 2009). The study showed that azole resistant *A. fumigatus* isolates were found in the clinical settings and the environment outside the hospitals, and the environmental and clinical isolates were genetically highly similar (Snelders et al., 2009).

One major finding of our study is that the *C. tropicalis* fluconazole resistant rate in candiduria group has notably increased from 6.8 to 29.5% during 2010–2011 to 2012–2013 (*p* = 0.001) and maintained at high level, whilst resistant rates in invasive infections group were significantly lower until 2019 (<15%), only after that an increase was observed in 2020–2021 (to 17.2%). There are two potential explanations on the origin and/or evolution of high azole resistance in candiduria *C. tropicalis* isolates. First, apart from being part of the human normal microbiota, *C. tropicalis* is also found in other animals and in wide-range of environment such was water and sands (Buck et al., 1977; Zuza-Alves et al., 2016, 2019). Therefore, it is possible that azole resistant *C. tropicalis* isolates originated from the natural environment spread to humans as colonizers and become a source of invasive infections. Second, azoles were still widely used in clinical treatment of fungal infections and as prophylaxis for high-risk populations (Vardakas et al., 2006; Hicheri et al., 2012; Coussement et al., 2021). Previous study indicated that immunosuppression coupled with antimicrobial stress could result in rapid selection of resistance and expansion of antimicrobial-resistant populations in the respiratory tract (Huo et al., 2022). Fluconazole is primarily renal cleared and has high urinary concentration (Lazar and Wilner, 1990), which could become a selective pressure for resistant isolates. We speculate that urinary tract environment might be a potential reservoir in the evolution and dissemination of azole resistance in *C. tropicalis*. Similar hypothesis on urinary environment as a potential source of antifungal resistance development was also put forward in other *Candida* species like *Candida auris* (Biagi et al., 2019; Asadzadeh et al., 2022).

In this study, four *ERG11* missense mutations, namely T374C (V125A), A395T/W (Y132F), C461T/Y (S154F) and T769C/Y (Y257H), were detected amongst fluconazole-resistant isolates, all of which have been well-characterized previously (Fan et al., 2020; Keighley et al., 2022; Leepattarakit et al., 2022). The A395T/W mutation at *ERG11*, which resulted in Y132F substitution, can cause a dramatic increase in the MIC values to azoles in *C. tropicalis* (Fan et al., 2019). In *Saccharomyces cerevisiae* functional verification system, Y132F substitution led to 5-fold increase of MICs towards azoles (Fan et al., 2019). Our sequencing data indicated that mutations A395T/W in *ERG11* are the major mechanism linked to azole resistance, which is consistent with previous findings (Fan et al., 2019). Moreover, the number of isolates with heterozygous mutation at position 395 has been increasing and become dominant in the later years of surveillance. However, the fluconazole MIC values of the heterozygous mutations (A395W) isolates equally high to those carrying homozygous mutations (A395T), suggesting that this amino acid substitution has a strong effect in causing azole-resistant phenotypes. However, there may be other mechanisms (Sasani et al., 2021; Won et al., 2021) that contribute to azole resistance in combination with the mutation of the target gene, which need to be further verified.

One of the limitations in our study was that *C. tropicalis* candiduria could not be unambiguously attributed to infection or colonization sources. Although in around 5% patients, *C. tropicalis* was identified simultaneously from both urinary and IVF samples, we did not perform genetic relatedness analysis for these isolates in the current study. In addition, the azole-resistant *C. tropicalis* isolates containing the A395W mutation should be further studied genetically to clarify the origin and dissemination pattern.

In conclusion, our study showed that the azole resistance in *C. tropicalis* in candiduria has reached a concerning high level and significantly prior to invasive isolates in our center. Urinary tract may be an important reservoir for acquiring, emerging, and spreading of azole-resistant *C. tropicalis*. Mutations A395T/W in *ERG11* are the major mechanism conferring azole resistance. In view of “One Health” issue, monitoring the azole resistant trend in *Candida* species causing candiduria and understanding its role is warrant in curbing antifungal resistance and for patient therapy and treatment.

## Data availability statement

The raw data supporting the conclusions of this article will be made available by the authors, without undue reservation. New *ERG11* gene sequences have been deposited in NCBI GenBank database under accession nos. OQ413996 and OQ413997.

## Author contributions

XF, Z-jL, and C-xY conceived the work. XF, XC, PW, Z-jL, and C-xY performed the experiments. XF and CT performed the data analysis and drafting the manuscript. All authors contributed to the article and approved the submitted version.

## Funding

This work was supported by the Natural Science Foundation of China (81802042) and Beijing Hospitals Authority Youth Program (QML20190301).

## Conflict of interest

The authors declare that the research was conducted in the absence of any commercial or financial relationships that could be construed as a potential conflict of interest.

## Publisher’s note

All claims expressed in this article are solely those of the authors and do not necessarily represent those of their affiliated organizations, or those of the publisher, the editors and the reviewers. Any product that may be evaluated in this article, or claim that may be made by its manufacturer, is not guaranteed or endorsed by the publisher.
