# Supplementary material for: High prevalence of fluconazole resistant Candida tropicalis among candiduria samples in China: An ignored matter of concern
Source: Front Microbiol. 2023 Mar 2;14:1125241. doi: 10.3389/fmicb.2023.1125241 (PMC10017723; doi:10.3389/fmicb.2023.1125241)
Supplement: Supplementary file 1 [file Data_Sheet_1.PDF]

Supplementary Table 1. Specimen distribution of *C. tropicalis* isolates detected in this study.

Specimen types	Year												Total
	**2010**	**2011**	**2012**	**2013**	**2014**	**2015**	**2016**	**2017**	**2018**	**2019**	**2020**	**2021**	
**Urine samples**	39	35	23	21	16	37	20	38	37	29	33	35	363
**Invasive infection samples**	10	7	9	11	16	7	11	21	16	18	15	14	155
Ascitic fluid	2	2	1	2	3	1	5	4	7	4	7	5	43
BALF	4		2	4	3	2	2	6	4	7	4	5	43
Bile	1	3	2	3	3	2	0	3	0	3	1		21
Pus	0	0	2	0	2	2	2	4	3	1	2	1	19
Blood	1	0	0	0	2	0	0	3	2	2	0	1	11
Pleural fluid	0	1	1	1	1	0	1	1	0	1	1	2	10
CVC	2	1	1	0	2	0	1	0	0	1	0	0	8
Tissue	0	0	0	1		0	0	0	0	0	0	0	1
**Total**	**49**	**42**	**32**	**32**	**32**	**44**	**31**	**59**	**53**	**48**	**48**	**49**	**519**

Abbreviations: BALF, bronchoalveolar lavage fluid; CVC, central venous catheter.
